# Fall risk and its determinants among home-dwelling older adults: A multivariate logistic regression analysis

**DOI:** 10.12669/pjms.42.4.12475

**Published:** 2026-04

**Authors:** Ozge Tuncer, Ayca Asma Sakalli, Nil Tekin

**Affiliations:** 1 Ozge Tuncer, MD. Associate Professor, Department of Family Medicine, Buca Seyfi Demirsoy Training and Research Hospital, Izmir, Turkey; 2 Ayca Asma Sakalli, MD, Specialist. Department of Family Medicine, Balikesir Ataturk City Hospital, Balikesir, Turkey; 3 Nil Tekin, MD. Associate Professor, Department of Family Medicine, Tepecik Training and Research Hospital, Izmir, Turkey

**Keywords:** Accidental falls, Fall prevention, Older people, Risk factors, Screening

## Abstract

**Objectives:**

Older adults receiving home healthcare are at increased risk of falls due to environmental, nutritional, psychological, and functional vulnerabilities. Fear of falling (FoF) may further restrict activity and contribute to adverse outcomes. This study aimed to examine factors associated with fall history and FoF using multivariate logistic regression analysis.

**Methods:**

A cross-sectional study was conducted among 320 older adults (≥65 years) receiving home healthcare at Izmir Bozyaka Training and Research Hospital between November 2023 and January 2024. Data were collected during home visits using validated tools: FRAIL Scale, Malnutrition Universal Screening Tool (MUST), Barthel Index, Itaki Fall Risk Scale, Six-Item Screener, Tinetti Balance and Gait Test, and Geriatric Depression Scale-Short Form (GDS-SF). Fall history and FoF were self-reported. Predictors were identified using multivariate logistic regression models.

**Results:**

The mean age was 81.52±7.57 years; 53.8% were female. Fall history and FoF were reported by 58.8% and 54.4%, respectively. Multivariate logistic regression analysis revealed that fall history was significantly predicted by environmental hazards (OR=37.37), age ≥80 (OR=2.60), high risk on Itaki (OR=12.15) and Tinetti (OR=5.44) scales, malnutrition risk (OR=4.64), and moderate/severe depression (OR=2.58). FoF was significantly associated with obesity (OR=5.84), calf circumference ≤31 cm (OR=3.55), environmental hazards (OR=8.90), malnutrition risk (OR=3.33), and moderate/severe depression (OR=3.66).

**Conclusion:**

Falls and fear of falling in home-dwelling older adults are multifactorial issues predominantly driven by environmental safety and functional mobility. Comprehensive geriatric assessments, focusing on home environment modifications and nutritional support, are essential to reduce fall-related morbidity in this population.

***Abbreviations:* BMI:** Body mass index, **CC:** Calf circumference, **FoF:** Fear of Falling, **GDS-SF:** Geriatric Depression Scale-Short Form, **HHS:** Home Healthcare Service, **MUST:** Malnutrition Universal Screening Tool, **SIS:** Six Item Screener.

## INTRODUCTION

The geriatric population is rapidly increasing in Turkey and globally, making independence and aging in place essential for healthy aging. Falls and fractures pose significant threats, with no reduction in fatalities or severe injuries despite extensive research over the past 15 years.^1^ Among geriatric syndromes, falls refer to accidental drops to the ground or a lower level, occurring 55% at home, 20% near home and 25% elsewhere, accounting for 40% of trauma-related hospitalizations. Consequences range from pain, bruising and fractures to severe cases like intracranial hemorrhage. In Turkey, fall prevalence is 35.4%.^2^

The 2019 Multidimensional Older Adult Monitoring Guide by the Turkish Ministry of Health recommends assessing fall risk by asking, *“Have you fallen in the past year?”* However, there is no nationally standardized, widely implemented evidence-based fall-prevention guideline specifically tailored for home healthcare settings in Turkey.^3^ Fear of falling (FoF), depression, loss of independence, hospitalizations and even mortality place a significant burden on the healthcare system. Many older adults do not report falls unless injured, underscoring the need for early screening and comprehensive assessments.^4^

Falls result from multiple risk factors, some modifiable and others not. The risk increases with the number of contributing factors.^5^ Therefore, this study aimed to determine the prevalence of fall history and fear of falling (FoF) among older adults receiving Home Health Services (HHS) and to identify their predictive factors using multivariate logistic regression models. Although falls and fear of falling have been widely studied in community-dwelling older adults, evidence remains limited for home healthcare recipients, who represent a frailer subgroup with a higher burden of malnutrition, depressive symptoms, and environmental hazards. Moreover, few studies have simultaneously examined fall history and fear of falling within the same multivariable framework in this setting.

## METHODOLOGY

Written informed consent was obtained from all participants or their legal representatives prior to data collection between November 2023 to January 2024, home visits were conducted for patients registered at the Home Health Services (HHS) clinic of Izmir Bozyaka Training and Research Hospital. A total of 609 individuals were visited during this three-month period.

### Ethical Approval:

This cross-sectional study adhered to the Declaration of Helsinki. Ethical approval was obtained from the Ethics Committee of Izmir Bozyaka Training and Research Hospital (Republic of Türkiye Ministry of Health Clinical Research Ethics Committee Database; Approval No: 2023/15-10, dated October 3, 2023).

### Measures:

A data collection form was developed based on literature review, including 11 sociodemographic and clinical questions (age, gender, weight, height, BMI, chronic diseases and regular medication use). The number of chronic conditions such as diabetes, cardiovascular, cerebrovascular, respiratory, renal, neurodegenerative diseases, arthritis and cancer was recorded and grouped as 1, 2, 3, or ≥4. Environmental hazards at home were evaluated during visits and included inadequate lighting, slippery floors, physical obstacles, lack of handrails, inappropriate footwear, steps inside the house and bathtub use.^6^ Environmental hazards were assessed by direct observation and categorized as present/absent.Fall history was determined by asking whether the participant had fallen in the past year. Polypharmacy was defined as concurrent use of five or more medications.^7^

Geriatric syndrome assessment included the FRAIL Scale for frailty status^8^ and the Itaki Falls Scale to classify fall risk as low (0-4) or high (≥5).^9^ Nutritional risk was assessed by the Malnutrition Universal Screening Tool (MUST).^10^ Functional status was measured using the Barthel Index (0-100; higher scores indicate greater independence).^11^ Depression was screened with the 15-item Geriatric Depression Scale-Short Form (0-4: none, 5-8: mild, 9-11: moderate, ≥12: severe).^12^ Mobility and fall risk were evaluated using the Tinetti Balance and Gait Test (scores: 25-28 low risk, 19-24 moderate, <19 high).^13^ Fear of Falling (FoF) was assessed with a yes/no question.^14^

### Inclusion & Exclusion criteria:

**Fig.1:**
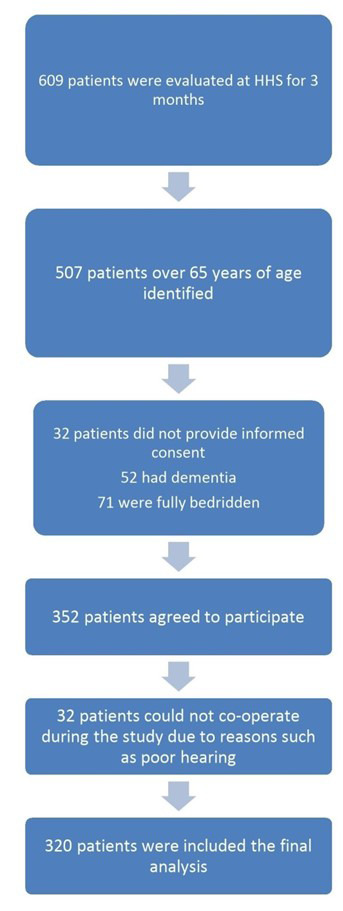
Flowchart of the study population.

Inclusion criteria were age ≥65, ability to respond to survey questions and enrollment in HHS. Patients <65 years, fully bedridden, or cognitively impaired were excluded. During a three months period, 609 home visits were conducted. Of these, 102 were under 65, 32 declined participations, 52 had dementia and 71 were bedridden. Additionally, 32 were excluded due to hearing problems. After exclusions, 320 patients were enrolled and assessed during home visits. Informed consent was obtained prior to participation (Fig.1).

### Statistical analysis:

Data were analyzed using IBM SPSS Statistics Version 27.0. Descriptive statistics included frequency, percentage, mean, standard deviation and median. To identify the independent predictive factors for fall history and fear of falling, multivariate logistic regression analyses were conducted. Dependent variables (fall history and fear of falling) were explicitly coded as binary outcomes (1 = Yes, 0 = No) to resolve any directional ambiguity. Independent categorical predictors were also coded accordingly (e.g., environmental hazards: 1 = Present, 0 = Absent; MUST: 1 = Moderate/High risk, 0 = Low risk). The models included age group, gender, frailty, depression level, malnutrition risk, balance/gait impairment, and home environmental hazards. The results of the logistic regression models were reported as Odds Ratios (OR) with 95% Confidence Intervals (CI). A p-value of <0.05 was considered statistically significant.

## RESULTS

The mean age of the participants was 81.52 years (SD = 7.57) and 53.8% were female. Among the participants, 57.2% had environmental hazards in the home, 58.8% had a history of falls and 54.4% reported fear of falling. Table-I shows the comparisons of participant characteristics according to fall history. Age, BMI, calf circumference, number of medications used, environmental hazards in the home, frailty status, fall risk, functional dependency, nutritional status and depression levels were significantly different between participants with and without a history of falls.

**Table-I:** Correlation between sociodemographic characteristics and total scores according to participants’ history of falls.

		Fall History		p
		Yes	No	
		N (%)	N (%)	
*Age(years)*		82.96±7.28	79.48±7.52	<0.001
Gender	Male	85(57.4)	63(42.6)	0.657
	Female	103 (59.9)	69 (40.1)	
Number of chronic diseases	<3	87(56.5)	67(43.5)	0.430
	≥3	101(60.8)	65(39.2)	
BMI(kg/m^2)^	<23	141(56.6)	108(43.4)	0.020
	23-27	17(89.5)	2(10.5)	
	27-30	3(75.0)	1(25.0)	
	>30	27(56.3)	21(43.8)	
Number of medications used	0-3	29(43.9)	37(56.1)	0.010
	4-7	130(64.7)	71(35.3)	
	≥8	29(54.7)	24(45.3)	
Environmental hazards in the home	Yes	162(88.5)	21(11.5)	<0.001
	No	26(19.0)	111(81.0)	
FRAIL scale	Nonfrail/Prefrail	8(20.5)	31(79.5)	<0.001
	Frail	180(64.1)	101(35.9)	
Itaki Fall Risk Scale	Low	2(7.4)	25(92.6)	<0.001
	High	186 (63.5)	107(36.5)	
Barthel Index	Severe dependency	119(63.3)	69(36.7)	<0.001
	Moderate dependency	41(58.6)	29(41.4)	
	Slight dependency	22(57.9)	16(42.1)	
	Independence	6(25.0)	18(75.0)	
Tinetti Balance & Gait Test	High risk	159 (63.6)	91(36.4)	0.001
	Moderate-low risk	29(41.4)	41(58.6)	
Malnutrition Universal Screening Tool	Low risk	105(49.3)	108(50.7)	<0.001
	Medium risk	66(81.5)	15(18.5)	
	High risk	17(65.4)	9(34.6)	
Geriatric Depression Scale-Short Form	Normal or mild depression	121(53.3)	106(46.7)	0.002
	Moderate-severe depression	67(72.0)	26(28.0)	

The comparison of participants’ sociodemographic and clinical characteristics based on fear of falling are shown in Table-II. Age, number of medications used, calf circumference, environmental hazards in the home, frailty, fall risk, functional dependency, nutritional risk and depression level were significantly associated with fear of falling.

**Table-II:** Correlations between participants’ sociodemographic characteristics and scale scores according to fear of falling.

	Fear of falling		p
	Yes	No	
	N (%)	N (%)	
Age(years)		82.56±7.39	80.28±7.61	0.007
Gender	Male	78(52.7)	70(47.3)	0.577
	Female	96(55.8)	76(44.2)	
Number of chronic diseases	<3	79(51.3)	75(48.7)	0.287
	≥3	95(57.2)	71(42.8)	
BMI (kg/m^2^)	<23	134(53.8)	115(46.2)	0.329
	23-27	14(73.7)	5(26.3)	
	27-30	2(50.0)	2(50.0)	
	>30	24(50.0)	24(50.0)	
Number of medications used	0-3	24(36.4)	42(63.6)	0.003
	4-7	121(60.2)	80(39.8)	
	≥8	29(54.7)	24(45.3)	
Calf circumference(cm)	≤31	63(82.9)	13(17.1)	<0.001
	>31	111(45.5)	133(54.5)	
Environmental hazards in the home	Yes	141(77.0)	42(23.0)	<0.001
	No	33(24.1)	104(75.9)	
FRAIL scale	Non or Prefrail	7(17.9)	32(82.1)	<0.001
	Frail	167(59.4)	114(40.6)	
Itaki Fall Risk Scale	Low	2(7.4)	25(92.6)	<0.001
	High	172(58.7)	121(41.3)	
Barthel Index	Severe dependency	115(61.2)	73(38.8)	<0.001
	Moderate dependency	39(55.7)	31(44.3)	
	Slight dependency	16(42.1)	22(57.9)	
	Independence	4(16.7)	20(83.3)	
Tinetti Balance & Gait Test	High risk	146(58.4)	104(41.6)	0.006
	Moderate-Low risk	28(40.0)	42(60.0)	
Malnutrition Universal Screening Tool	Low risk	95(44.6)	118(55.4)	<0.001
	Medium risk	61(75.3)	20(24.7)	
	High risk	18(69.2)	8(30.8)	
Geriatric Depression Scale-Short Form	Normal or mild depression	106(46.7)	121(53.3)	<0.001
	Moderate-severe depression	68(73.1)	25(26.9)	

Predictors of fall history: Multivariate logistic regression analysis was performed to identify the independent predictors of fall history. The model revealed that the presence of environmental hazards at home was the strongest predictor, increasing the odds of having a fall history by over 37 times (OR=37.37, 95% CI: 17.10-81.67, p<0.001). Other significant independent predictors included being ≥80 years of age (OR=2.60, 95% CI: 1.22-5.54, p=0.013), being at high risk according to the Itaki Fall Risk Scale (OR=12.15, 95% CI: 1.36-108.44, p=0.025), and high risk on the Tinetti Balance & Gait Test (OR=5.44, 95% CI: 1.96-15.08, p=0.001). Furthermore, moderate or high malnutrition risk according to MUST (OR=4.64, 95% CI: 1.57-13.70, p=0.005) and moderate or severe depression according to GDS-SF (OR=2.58, 95% CI: 1.11-5.99, p=0.028) significantly predicted fall history. Other variables such as BMI, calf circumference, polypharmacy, FRAIL, and Barthel Index were not statistically significant (p>0.05) (Table III).

**Table-III:** Predicting falling history with parameters

Parameters	β	S.E.	Wald	df	p value	Odds Ratio	95% C.I. for Odds Ratio / EXP(β)	
							Lower	Upper
Age (≥80 vs. <80)	0.95	0.39	6.11	1	0.013	2.60	1.22	5.54
BMI Groups			2.19	3	0.534			
BMI Groups (obese vs. underweight)	-0.61	0.51	1.41	1	0.234	1.84	0.67	5.03
BMI Groups (obese vs. normal)	0.14	1.12	0.02	1	0.899	1.15	0.13	10.26
BMI Groups (obese vs. overweight)	-0.03	1.43	0.00	1	0.984	1.03	0.06	16.98
Calf circumference (>31 vs. ≤31 cm)	0.64	0.47	1.91	1	0.167	1.90	0.76	4.74
Polypharmacy (≥5 vs. <5)	-0.26	0.56	0.21	1	0.644	1.29	0.43	3.84
Environmental hazards in the home (Yes vs. No)	3.62	0.40	82.41	1	<0.0001	37.37	17.10	81.67
FRAIL (Frail vs. Nonfrail/Prefrail)	0.49	0.44	1.26	1	0.262	1.63	0.69	3.86
Itaki Fall Risk Scale (High vs. Low)	2.50	1.12	5.00	1	0.025	12.15	1.36	108.44
Barthel Index			1.27	3	0.737			
Barthel Index (Severe dependency vs. Moderate dependency)	0.22	0.46	0.24	1	0.626	1.25	0.51	3.09
Barthel Index (Severe dependency vs. Slight dependency)	0.63	0.68	0.85	1	0.355	1.88	0.49	7.18
Barthel Index (Severe dependency vs. Independence)	0.66	0.80	0.67	1	0.414	1.93	0.40	9.31
Tinetti Balance & Gait Test (High vs. Moderate&Low Risk)	1.69	0.52	10.58	1	0.001	5.44	1.96	15.08
MUST (High & Moderate Risk vs. Low risk)	1.53	0.55	7.73	1	0.005	4.64	1.57	13.70
GDS-SF (Moderate-severe vs. Normal or mild depression)	0.95	0.43	4.86	1	0.028	2.58	1.11	5.99
Constant	-1.58	0.86	3.40	1	0.065	4.86		

BMI: Body Mass Index. GDS-SF: Geriatric Depression Scale-Short Form. MUST: Malnutrition Universal Screening Tool.

Logistic regression analysis and enter method used. p<0.05 considered significant.

### Predictors of Fear of Falling (FoF):

A separate multivariate logistic regression model was established for fear of falling. The analysis identified environmental hazards at home as a major predictor (OR=8.90, 95% CI: 4.79-16.55, p<0.001). Additionally, obesity (BMI Obese vs. Normal; OR=5.84, 95% CI: 1.06-32.12, p=0.043), having a calf circumference of $\le$31 cm (OR=3.55, 95% CI: 1.62-7.81, p=0.002), moderate or high malnutrition risk (MUST; OR=3.33, 95% CI: 1.41-7.88, p=0.006), and moderate or severe depression (GDS-SF; OR=3.66, 95% CI: 1.75-7.66, p=0.001) were significant independent predictors of FoF. Age, polypharmacy, FRAIL, Itaki, Barthel, and Tinetti scales were not significant predictors in this model (p>0.05) (Table IV).

**Table-IV:** Predicting fear of falling risk with parameters

Parameters	β	S.E.	Wald	df	p value	Odds Ratio	95% C.I. for Odds Ratio / EXP(β)	
							Lower	Upper
Age (≥80 vs. <80)	0.47	0.31	2.27	1	0.132	1.60	0.87	2.94
BMI Groups			5.01	3	0.171			
BMI Groups (obese vs. underweight)	0.54	0.42	1.65	1	0.199	1.71	0.75	3.87
BMI Groups (obese vs. normal)	1.76	0.87	4.11	1	0.043	5.84	1.06	32.12
BMI Groups (obese vs. overweight)	2.02	1.25	2.59	1	0.108	7.51	0.64	87.54
Calf circumference (≤31 vs. >31 cm)	1.27	0.40	9.95	1	0.002	3.55	1.62	7.81
Polypharmacy (≥5 vs. <5)	0.58	0.43	1.80	1	0.179	1.78	0.77	4.13
Environmental hazards in the home (Yes vs. No)	2.19	0.32	47.71	1	<0.0001	8.90	4.79	16.55
FRAIL (Frail vs. Nonfrail/Prefrail)	0.28	0.36	0.63	1	0.426	1.33	0.66	2.67
Itaki Fall Risk Scale (High vs. Low)	1.76	0.93	3.60	1	0.058	5.82	0.94	35.90
Barthel Index			2.03	3	0.565			
Barthel Index (Severe dependency vs. Moderate dependency)	0.13	0.38	0.11	1	0.735	1.14	0.54	2.40
Barthel Index (Severe dependency vs. Slight dependency)	-0.58	0.51	1.30	1	0.255	1.78	0.66	4.80
Barthel Index (Severe dependency vs. Independence)	-0.48	0.74	0.42	1	0.515	1.62	0.38	6.92
Tinetti Balance & Gait Test (High vs. Moderate & Low Risk)	0.72	0.43	2.77	1	0.096	2.06	0.88	4.82
MUST (High & Moderate Risk vs. Low risk)	1.20	0.44	7.51	1	0.006	3.33	1.41	7.88
GDS-SF (Moderate-severe vs. Normal or mild depression)	1.30	0.38	11.86	1	0.001	3.66	1.75	7.66
Constant	0.21	0.74	0.08	1	0.781	1.23		

BMI: Body Mass Index, GDS-SF: Geriatric Depression Scale-Short Form, MUST: Malnutrition Universal Screening Tool.

Logistic regression analysis and enter method used. p<0.05 considered significant.

**Fig.2:**
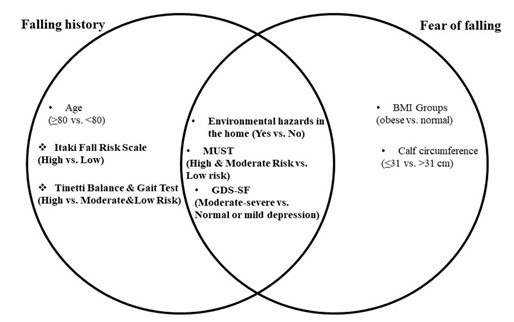
Common predictive parameters for fall history and fear of falling.

When comparing both models, environmental hazards at home, malnutrition risk (MUST), and depression (GDS-SF) emerged as common significant predictive factors for both fall history and fear of falling. These commonalities, alongside the unique predictors identified for each outcome, are visually summarized in the Venn diagram (Fig.2).

## DISCUSSION

This study is one of the few to simultaneously examine factors associated with both fall history and fear of falling (FOF) among older adults receiving home healthcare services. Using multivariate logistic regression models, the study holistically elucidates how environmental, nutritional, psychological, and functional factors independently predict fall-related outcomes in this vulnerable population.

The average participant age was 82 and both fall history and fear of falling (FOF) were highly prevalent. This aligns with prior research that links aging-related physiological changes-such as reduced muscle strength and slower reaction time-with increased fall risk.^15^,^16^ In our regression models, advanced age (≥ 80 years) significantly increased the odds of experiencing a fall by 2.6 times, underscoring the multifactorial nature of aging where physical changes contribute directly to vulnerability. However, age was not an independent predictor of FoF, which suggests that psychological fear is driven more by other modifiable factors than chronological age alone. This is consistent with findings that age’s influence on FoF is often mediated by decreased physical performance.^17^

Among all variables, environmental hazards in the home (EHH) were the strongest predictor of both outcomes (increasing the odds of falls by over 37 times, and FoF by nearly nine times). The magnitude of this association may partly reflect the high prevalence and clustering of multiple hazards in the home-care population, as well as the binary (present/absent) categorization of environmental risk, which may act as a strong discriminator between individuals with and without fall-related outcomes. This emphatically highlights the immense risks faced by older adults who spend most of their time at home. Home safety assessments and environmental modifications are paramount interventions, though their success may depend on the individual’s functional capacity. 18,19 Malnutrition risk, assessed via the MUST scale, was moderately associated with both fall history and FOF. Malnourished individuals often experience loss of muscle mass and physical frailty. Several studies have linked poor nutrition with lower physical performance, greater sarcopenia and impaired gait.^20^,^21^ Malnutrition may also increase FOF by contributing to inactivity and social withdrawal, reinforcing the need for nutritional screening in home healthcare settings.

Depressive symptoms (moderate to severe) were another common independent predictor, significantly increasing both fall history and FoF. Depression can severely reduce motivation and activity, increasing FoF through avoidance behaviors. Social isolation among homebound older adults may amplify this effect. Prior research robustly supports a bidirectional relationship between depression and FoF.^17^,^22^,^23^ Mobility impairments, measured by the Tinetti Balance and Gait Test, significantly predicted fall history. Although both the Itaki Fall Risk Scale and the Tinetti Balance and Gait Test are used to assess fall risk, they reflect different constructs. The Itaki scale captures a broader clinical and environmental risk profile, whereas the Tinetti test provides an objective measure of balance and gait performance. Therefore, including both tools in the multivariable model allowed us to account for complementary dimensions of fall-related vulnerability. Limited mobility raises actual fall risk, and interventions that maintain functional movement and confidence may help mitigate these risks. This is supported by Rehman et al., which demonstrated that targeted exercises significantly improve balance and reduce fall risk, reinforcing the need for early mobility interventions.^24^ This is also supported by findings that show FoF’s impact on quality of life is often mediated by physical performance.^25^

Unlike some prior studies, gender, polypharmacy, and frailty (FRAIL scale) were not significant independent predictors in our multivariate models. This may reflect the characteristics of the home healthcare population, where variations in support and health status could influence results.^17^,^22^ It’s also likely that depression, malnutrition and mobility issues mediate these relationships. Consistent with this multifactorial perspective, Alamri et al. also highlighted the significant correlations between falls and various geriatric syndromes in community-dwelling older adults, emphasizing the interconnected nature of these risks.^26^

### Strengths and Limitations:

The strengths of this study include its comprehensive and holistic assessment of multiple geriatric syndromes simultaneously within a home-care setting. The use of robust multivariate logistic regression models allowed us to meticulously adjust for confounders and identify independent predictive factors for both actual falls and the psychological fear of falling. However, the study has certain limitations. Its cross-sectional design restricts definitive causal inferences. Additionally, the reliance on self-reported data for fall history may introduce recall bias, and the findings may have limited generalizability beyond home-healthcare populations. Future longitudinal studies and randomized controlled interventional trials focusing on the common predictors identified here specifically environmental modifications, nutritional support, and psychological care are urgently needed to establish causality and guide targeted fall-prevention strategies.

## CONCLUSIONS

This study demonstrates that both fall history and fear of falling among home-dwelling older adults are independently predicted by shared modifiable risk factors, predominantly environmental hazards at home, malnutrition risk, and depressive symptoms. Additionally, actual fall history is strongly associated with advanced age and impaired balance, whereas fear of falling is further influenced by physical factors such as obesity and reduced calf circumference. These findings underscore the critical need for multidimensional, targeted interventions specifically home safety modifications, proactive nutritional screening, psychological support, and mobility enhancement to effectively mitigate fall risks. Addressing these interconnected geriatric syndromes holistically can significantly improve the safety, independence, and overall quality of life of older adults receiving home healthcare.

### Declaration of AI Use:

The authors declare that an AI assisted language tool was used solely for English language editing and grammar improvement to enhance the readability of the manuscript. The scientific content, conceptualization, data interpretation, and final manuscript drafting were performed entirely by the authors without the use of any AI tools.

### Authors’ contributions:

**OT:** Conceptualization, Methodology, Supervision, writing original draft, review & editing, responsible and accountable for the accuracy and integrity of the entire work.

**AAS:** Investigation, Writing - original draft, Writing - review & editing.

**NT:** Methodology, Supervision.

All authors have read and approved the final version of the manuscript.
